# Case series and Literature review – Clozapine Induced Transient Myocarditis. Clinical characteristics and outcomes

**DOI:** 10.1192/j.eurpsy.2024.508

**Published:** 2024-08-27

**Authors:** U. L. Anyeji, P. Bidkhanian

**Affiliations:** Psychiatry, BronxCare Health System, Bronx, United States

## Abstract

**Introduction:**

Clozapine is a second-generation atypical antipsychotic medication used in patients with treatment-refractory schizophrenia. Its use is limited due to its associated adverse effects, including myocarditis. These adverse effects may have variable presentations, such as myocarditis transient or persistent, and a generalized inflammatory process. Thus, clinical monitoring to inform accurate diagnosis is essential to avoid unnecessary discontinuation of clozapine, leading to psychiatric decompensation.

**Objectives:**

To review clinical features of clozapine induced Myocarditis and accurately identify signs and symptoms attributed to be most specific for myocarditis and determine at what stage clozapine should be discontinued.

**Methods:**

We conducted a literature review on PubMed, MeSH, google scholar and Mount Sinai’s Levy Library using keywords, clozapine, drug related side effects, adverse reaction, myocarditis, treatment resistant schizophrenia. Review of two cases series was done.

**Results:**

A review of 15 articles that addressed the cardiac complications of clozapine was performed. This review provides a base on variable clinical characteristics and outcomes of clozapine –induced Myocarditis. It showed patients who had myocarditis ruled out, demonstrated high prevalence of systemic signs of inflammation such as fever, malaise, tachycardia and elevated c-reactive protein. However, despite clozapine maintenance in most, this systemic response subsided without any intervention. A nonspecific inflammatory response is common when initiating clozapine, this inflammatory “clozapine storm’ occurs within the first month of initiation and is not necessarily predictive of myocarditis. These patients were monitored closely. Those confirmed with clozapine- induced myocarditis using echocardiography and cardiac magnetic resonance imaging were managed with dose reduction, laboratory monitoring, vital signs check, with early initiation of beta-blockers without discontinuation of clozapine, with improvement in their laboratory results and vital signs. Those with progressive clinical signs of myocarditis required immediate cessation of clozapine.

**Conclusions:**

We are proposing a critical need for a multidisciplinary team of psychiatrists, cardiologists and pharmacists collaborating to prevent premature termination of clozapine in cases of treatment-refractory schizophrenia. Our cases showed middle aged patients with treatment - refractory schizophrenia, presenting with symptoms suggestive of clozapine induced- myocarditis, few weeks after initiation. Clozapine was continued with close monitoring, as symptoms resolved. Though clozapine is associated with myocarditis, with proper knowledge on guidelines for monitoring patients, it can mitigate unnecessary discontinuation of clozapine in those patients.

**Disclosure of Interest:**

None Declared

